# The role of hexose transporter-like sensor hxs1 and transcription activator involved in carbohydrate sensing azf1 in xylose and glucose fermentation in the thermotolerant yeast *Ogataea polymorpha*

**DOI:** 10.1186/s12934-022-01889-z

**Published:** 2022-08-13

**Authors:** Marta V. Semkiv, Justyna Ruchala, Aksynia Y. Tsaruk, Anastasiya Z. Zazulya, Roksolana V. Vasylyshyn, Olena V. Dmytruk, MingXing Zuo, Yingqian Kang, Kostyantyn V. Dmytruk, Andriy A. Sibirny

**Affiliations:** 1 Institute of Cell Biology, NAS of Ukraine, Drahomanov St, 14/16, 79005 Lviv, Ukraine; 2 University of Rzeszow, Zelwerowicza 4, 35-601 Rzeszow, Poland; 3 State Key Laboratory of Functions and Applications of Medicinal Plants, Guizhou Medical University, Guizhou 550014 Guiyang, China; 4 Department of Microbiology, School of Basic Medical Sciences, Guizhou Medical University, Guizhou 550014 Guiyang, China

**Keywords:** Fuel ethanol, Yeast *Ogataea polymorpha*, Lignocellulose, Xylose, Alcoholic fermentation, Sensors

## Abstract

**Background:**

Fuel ethanol from lignocellulose could be important source of renewable energy. However, to make the process feasible, more efficient microbial fermentation of pentose sugars, mainly xylose, should be achieved. The native xylose-fermenting thermotolerant yeast *Ogataea polymorpha* is a promising organism for further development. Efficacy of xylose alcoholic fermentation by *O. polymorpha* was significantly improved by metabolic engineering. Still, genes involved in regulation of xylose fermentation are insufficiently studied.

**Results:**

We isolated an insertional mutant of *O.* *polymorpha* with impaired ethanol production from xylose. The insertion occurred in the gene *HXS1* that encodes hexose transporter-like sensor, a close homolog of *Saccharomyces cerevisiae* sensors Snf3 and Rgt2. The role of this gene in xylose utilization and fermentation was not previously elucidated. We additionally analyzed *O.* *polymorpha* strains with the deletion and overexpression of the corresponding gene. Strains with deletion of the *HXS1* gene had slower rate of glucose and xylose consumption and produced 4 times less ethanol than the wild-type strain, whereas overexpression of *HXS1* led to 10% increase of ethanol production from glucose and more than 2 times increase of ethanol production from xylose. We also constructed strains of *O.* *polymorpha* with overexpression of the gene *AZF1* homologous to *S. cerevisiae AZF1* gene which encodes transcription activator involved in carbohydrate sensing. Such transformants produced 10% more ethanol in glucose medium and 2.4 times more ethanol in xylose medium. Besides, we deleted the *AZF1* gene in *O. polymorpha*. Ethanol accumulation in xylose and glucose media in such deletion strains dropped 1.5 and 1.8 times respectively. Overexpression of the *HXS1* and *AZF1* genes was also obtained in the advanced ethanol producer from xylose. The corresponding strains were characterized by 20–40% elevated ethanol accumulation in xylose medium. To understand underlying mechanisms of the observed phenotypes, specific enzymatic activities were evaluated in the isolated recombinant strains.

**Conclusions:**

This paper shows the important role of hexose sensor Hxs1 and transcription factor Azf1 in xylose and glucose alcoholic fermentation in the native xylose-fermenting yeast *O. polymorpha* and suggests potential importance of the corresponding genes for construction of the advanced ethanol producers from the major sugars of lignocellulose.

## Background

Pentose metabolism and bioconversion to biofuels and high-value chemicals is important biotechnological problem attracting a large number of scientists. Most attention has been paid to metabolism and alcoholic fermentation of xylose, the most abundant pentose and the second monosaccharide on the planet after glucose. As a rule, xylose is a major component of hemicelluloses whereas free xylose is rarely found. Thus, many microorganisms cannot metabolize xylose and those which do, as a rule accumulate only tiny amounts of ethanol from xylose. Baker’s yeast *Saccharomyces cerevisiae* does not metabolize xylose though xylose-fermenting strains have been constructed [1]. The natural xylose-metabolizing yeasts like *Spathaspora passalidarum, Scheffersomyces stipitis* and others attract interest of many researchers [2, 3]. Among native xylose-fermenting yeasts, thermotolerant species are of special interest as they in principle are suitable for the use in simultaneous saccharification and fermentation (SSF) process. Two such species are under development, *Kluyveromyces marxianus* [4] and *Ogataea polymorpha*; the authors of this paper have been working for a while with the latter [5, 6]. Wild-type strains of *O. polymorpha* show a robust growth on both glucose and xylose, however, accumulate nearly 200 times less ethanol from xylose than from glucose [7]. Using methods of metabolic engineering and classical selection, recombinant strains of *O. polymorpha* have been constructed which accumulate at elevated temperature of 45 °C increased for 40 times amounts of ethanol from xylose as compared to the wild-type strain [8–14]. It was found that transcription factors and sugar transporters play an important role in regulation of xylose alcoholic fermentation. The role of sugar sensors in the process of xylose fermentation has not been studies yet. Earlier, we have described hexose transporter-like sensor gene *HXS1* in *O.* *polymorpha* [15]. This gene was not involved in glucose repression or catabolite inactivation though its deletion led to significantly impaired transient transcriptional repression in response to fructose. The role of this gene in xylose metabolism and in glucose and xylose alcoholic fermentation has not been studied. The current article is devoted to studying the role of the hexose sensor gene *HXS1* and the gene *AZF1*, encoding for *O.* *polymorpha* homolog of *S. cerevisiae* transcription factor with sensing properties, in xylose and glucose alcoholic fermentation in *O. polymorpha*. It was found that both genes act as activators of xylose and glucose fermentation as their knock outs impair sugar fermentation, whereas overexpression, inversely, activates this process. It is important to note that overexpression of both *HXS1* and *AZF1* on the background of the advanced ethanol producer further improves ethanol production from xylose which could be of biotechnological interest.

## Results

**Fig. 1:**
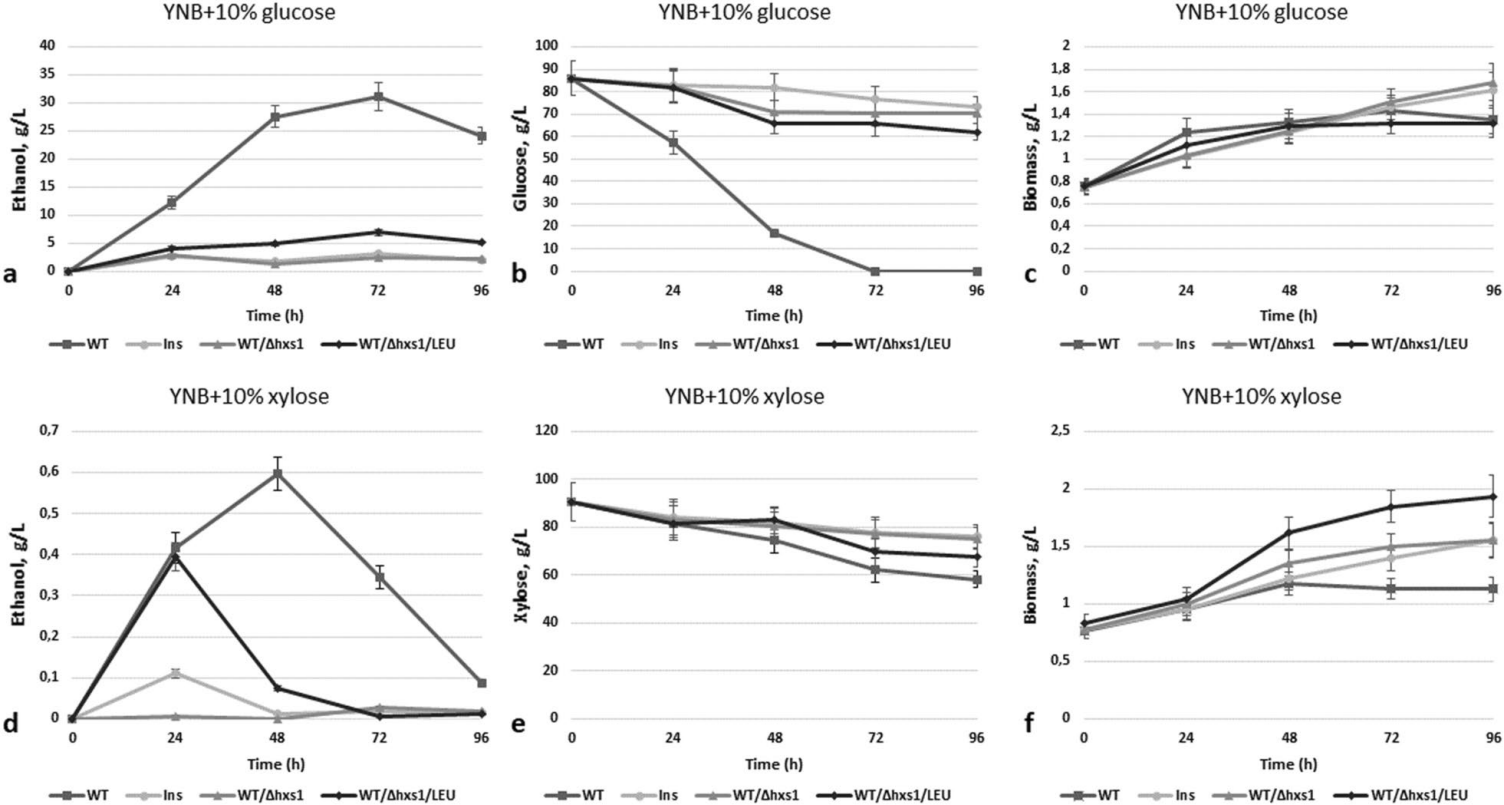
Ethanol production **a, d**, sugar consumption **b, e** and biomass accumulation **c, f** during 10% glucose and 10% xylose alcoholic fermentation of *O. polymorpha* wild type and recombinant strains with *HXS1* gene deletion at 37 °C. Data are shown as mean of three independent experiments

In efforts to tag the genes involved in regulation of xylose alcoholic fermentation, we applied method of insertion mutagenesis to search for the mutants with modified (elevated or decreased) ethanol production from xylose. One of such mutants, Ins, was taken for a detailed analysis. This strain almost did not produce any ethanol from xylose and its ability to produce ethanol from glucose was severely diminished—it produced 15 times less ethanol from glucose in comparison to the WT strain (Fig. 1a, d).

**Fig. 2:**
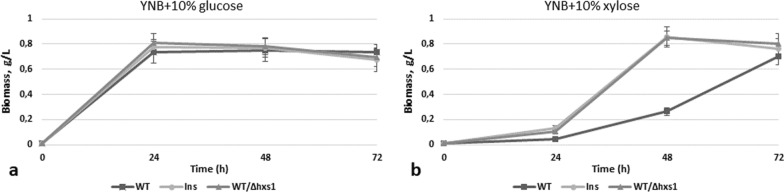
Growth test of the *O. polymorpha* wild type and recombinant strain with disruption of *HXS1* gene on different media, cultivation at 37 °C

DNA sequencing of the isolated insertion cassette with flanking sequences showed that insertion occurred in the previously identified by us gene *HXS1* encoding for transporter-like hexose sensor [15]. In order to confirm that observed phenotype of the insertion mutant (Ins) was caused by the disruption of *HXS1*, this gene was deleted in the wild-type *O. polymorpha* strain NCYC495. Obtained deletion mutant was named WT/Δhxs1. Also, for comparison, we took strain WT/Δhxs1/LEU, where *HXS1* deletion was obtained using a heterologous selection marker *ScLEU2*, meaning that prototrophy for leucine was restored in this strain. This strain was isolated by us previously [15] (in that article it was mentioned as Hp015). It was found that Ins as well as deletion strains were characterized by a severe drop in ethanol production from glucose—they produced only up to 7 g/L of ethanol during glucose alcoholic fermentation, whereas WT strain (NCYC495) produced up to 31 g/L of ethanol (Fig. 1a). Also, all strains with the disrupted gene *HXS1* almost did not produce any ethanol from xylose—0–0.1 g/L, with the exception of WT/Δhxs1/LEU strain producing 0.4 g/L of ethanol on 24 h of xylose fermentation (Fig. 1d). It was also shown that the Ins strain with disruption of the *HXS1* gene grew better than the WT strain in the medium with xylose, but not in the medium with glucose as a sole Carbon source (Fig. 2a, b).

Although the obtained data suggested that Hxs1 sensor is necessary for efficient fermentation of glucose and xylose, it could not be excluded that this sensor is a limiting factor in sugar uptake and subsequent intracellular metabolism. Indeed, strains with deletion of the *HXS1* gene were characterized by a slower consumption of xylose (Fig. 1e) and much slower consumption of glucose (Fig. 1b) during alcoholic fermentation as compared to the WT strain. Improved growth of Ins mutant on xylose could be explained if deletion mutants switch faster from glucose to xylose metabolism than strains with an intact Hxs1 protein.

**Fig. 3:**
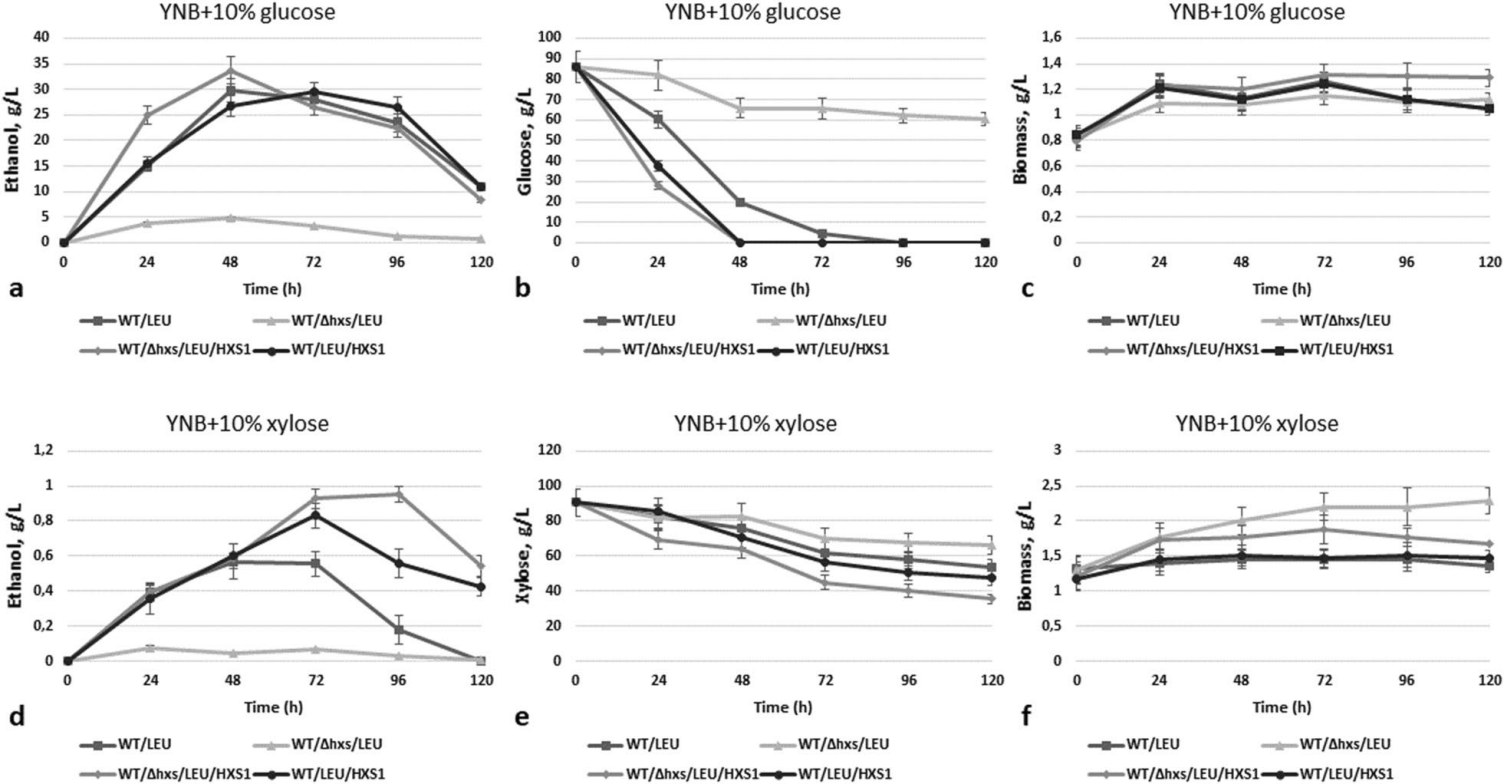
Ethanol production **a, d**, sugar consumption **b, e** and biomass accumulation **c, f** during 10% glucose and 10% xylose alcoholic fermentation of *O. polymorpha* wild type and recombinant strains with *HXS1* gene deletion and/or overexpression at 37 °C. Data are shown as mean of three independent experiments

We further decided to overexpress the *HXS1* gene under control of strong constitutive *GAP1* promoter of the glyceraldehyde-3-phosphate dehydrogenase gene and to study properties of the resulted transformants. For correct comparison, in all strains prototrophy for leucine was restored. It was observed that overexpression of the *HXS1* gene activated both glucose and xylose sugar fermentation when *HXS1* was overexpressed on the background of WT/LEU strain, as well as WT/Δhxs1/LEU strain, but the effect was more pronounced (up to 34 g/L of ethanol from glucose and 0.93 g/L of ethanol from xylose) when the overexpression was carried out on the background of deletion strain WT/Δhxs1/LEU (Fig. 3a, d). Strains with *HXS1* gene overexpression WT/Δhxs1/LEU/HXS1 and WT/LEU/HXS1 consumed all glucose in the medium in 48 h, whereas it took more than 72 h for parental WT strain to completely exhaust glucose (Fig. 3b). Also these strains consumed xylose faster than the WT strain and especially relative to strain with the *HXS1* gene deletion (Fig. 3e).

**Fig. 4:**
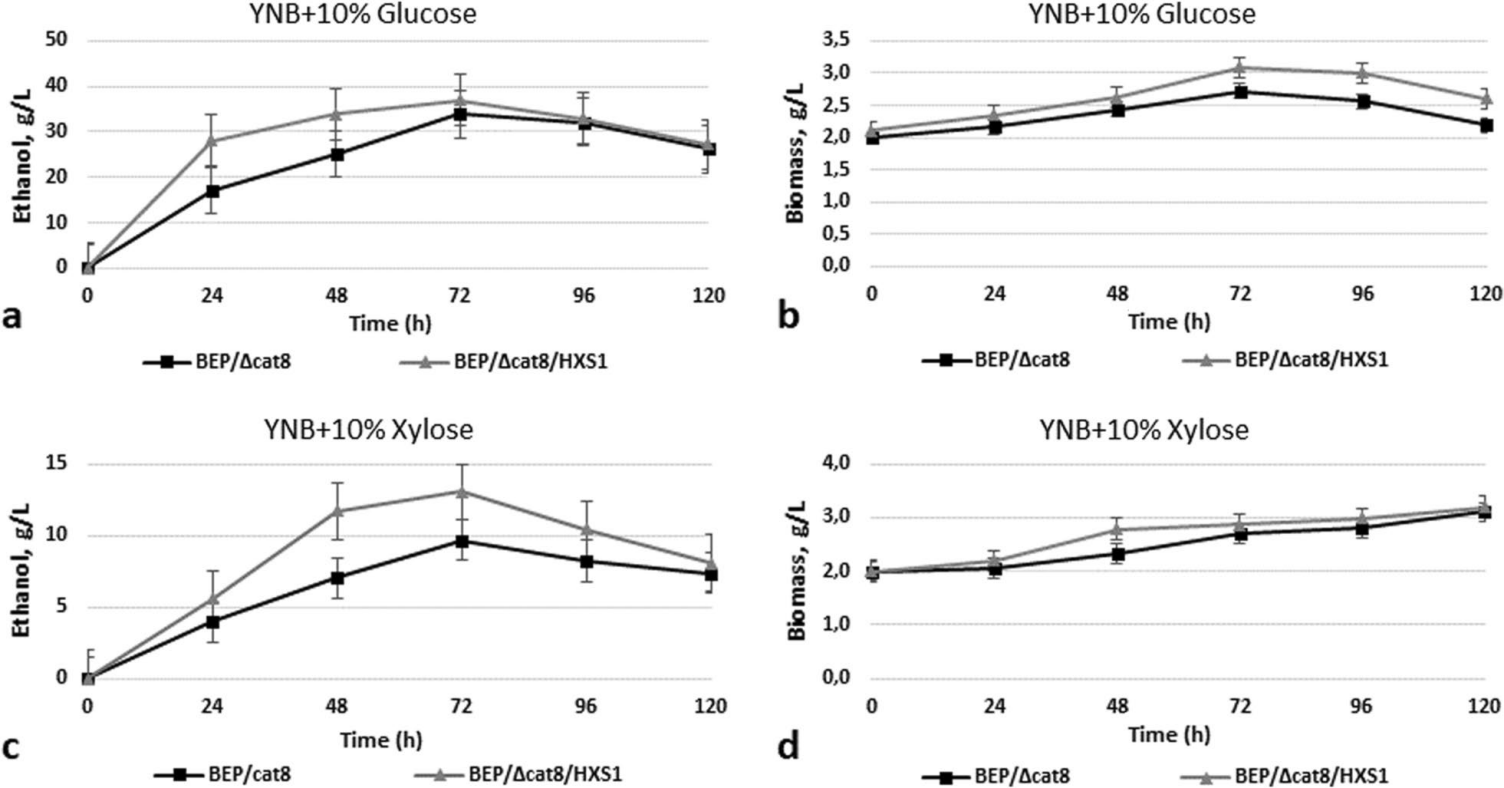
Ethanol production **a, c** and biomass accumulation **b, d** during 10% glucose and 10% xylose alcoholic fermentation of *O. polymorpha* wild type BEP/∆cat8 and recombinant strains with *HXS1* gene overexpression at 37 °C. Data are shown as mean of three independent experiments

Finally, we overexpressed the *HXS1* gene in the advanced ethanol producer from xylose (strain BEP/Δcat8) isolated by us earlier using several approaches of metabolic engineering and classical selection [11, 13]. Hxs1 overexpression in strain BEP/Δcat8/HXS1 activated ethanol production from glucose (37 g/L of ethanol against 34 g/L by strain BEP/Δcat8 on 72 h of fermentation) and from xylose (13 g/L of ethanol against 9.1 g/L by strain BEP/Δcat8 on 72 h of fermentation) (Fig. 4a, c, Table 1). One may suggest that Hxs1 sensor limits xylose uptake and utilization under conditions of activation of intracellular enzymes involved in xylose metabolism and fermentation, and therefore its overexpression enhances ethanol production.

**Table 1:** Main parameters of xylose fermentation at 37 °C by the tested *O. polymorpha* strains with *HXS1* and *AZF1* genes deletion and overexpression

Strain	Ethanol (g/L)	Ethanol yield (mg/g of consumed xylose)	Ethanol specific production rate (mg/g biomass/h)	Ethanol productivity (mg/L/h)
WT^**b**^	0.49 ± 0.03	15 ± 2	4 ± 1	7 ± 1
WT/LEU^**b**^	0.56 ± 0.07	19 ± 2	5 ± 1	8 ± 1
Ins^**b**^	0.02 ± 0.00	1 ± 0	0 ± 0	0 ± 0
WT/Δhxs1^**b**^	0.03 ± 0.00	2 ± 0	0 ± 0	0 ± 0
WT/Δhxs1/LEU^**b**^	0.01 ± 0.00	0 ± 0	0 ± 0	0 ± 0
WT/Δhxs/LEU/HXS1^**b**^	0.93 ± 0.06	20 ± 2	7 ± 1	13 ± 2
WT/LEU/HXS1^**b**^	0.83 ± 0.07	25 ± 4	8 ± 1	12 ± 3
WT/AZF1^**a**^	1.20 ± 0.05	41 ± 8	10 ± 3	25 ± 2
WT/∆azf1^**b**^	0.33 ± 0.03	20 ± 4	3 ± 1	4 ± 1
BEP/∆cat8^**b**^	9.10 ± 0.10	154 ± 20	49 ± 11	126 ± 22
BEP/∆cat8/AZF1^**c**^	11.02 ± 0.11	354 ± 31	40 ± 19	114 ± 19
BEP/∆cat8/HXS1^**b**^	13.04 ± 0.12	352 ± 29	63 ± 17	181 ± 27

^a^Data of ethanol yield (mg/g of consumed xylose), ethanol specific production rate (mg/g biomass/h), ethanol productivity (mg/L/h) and ethanol concentration (g/L) are represented on YNB medium supplemented with 10% of xylose on 48 h of fermentation

^b^72 h of fermentation

^c^96 h of fermentation

The *S. cerevisiae AZF1* gene codes for transcription activator of the genes involved in carbon metabolism and energy production on glucose. *S. cerevisiae azf1Δ* mutant cannot grow on glycerol. This transcription factor as [*AZF1*^+^] conformer was shown to form a prion [16–19]. In xylose-metabolizing *S. cerevisiae,* overexpression of *AZF1* increased rates of growth, xylose consumption, and ethanol production, but only when cells were anaerobically grown on xylose. In contrast, deletion of *AZF1* decreased growth and sugar fermentation, largely specific to anaerobic xylose growth [20, 21]. Thus *AZF1* was identified as sugar- and oxygen-responsive transcription factor. The role of the *AZF1* gene in xylose fermentation in the native xylose-metabolizing yeasts has never been studied previously. Here, we decided to elucidate the role of the *AZF1* homolog in xylose fermentation in *O.* *polymorpha*.

**Fig. 5:**
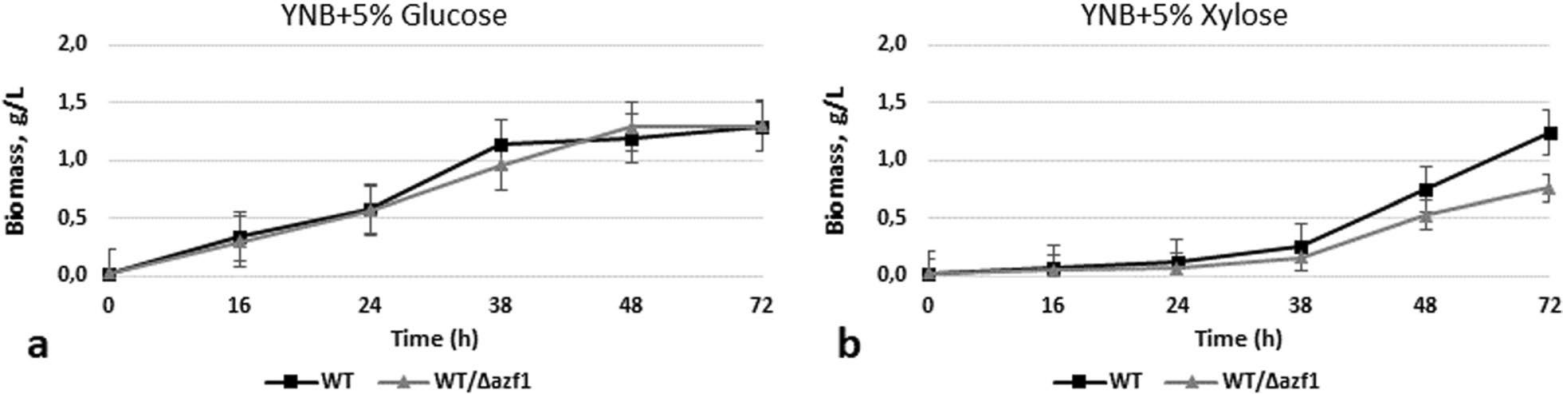
Growth test of the *O. polymorpha* wild type and recombinant strains with *AZF1* gene deletion on different media, cultivation at 37 °C

**Fig. 6:**
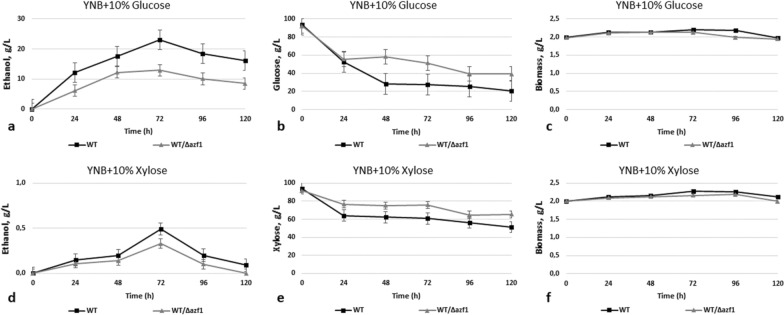
Ethanol production **a, d**, sugar consumption **b, e** and biomass accumulation **c, f** during 10% glucose and 10% xylose alcoholic fermentation of *O. polymorpha* wild type and recombinant strains with *Δazf1* gene deletion at 37 °C. Data are shown as mean of three independent experiments

The *O. polymorpha AZF1* gene homolog is much shorter than its counterpart from *S. cerevisiae* (1911 vs 2745 bp, respectively); the deduced proteins exhibit 26.5% identity and 34.7% similarity. We deleted the *AZF1* gene in *O. polymorpha* and overexpressed it on the backgrounds of the wild-type strain and of the advanced ethanol producer from xylose (strain BEP/Δcat8) [13]. It was found that WT/Δazf1 mutant is characterized by 40% decrease in growth on xylose at 72 h, whereas growth on glucose was not impaired (Fig. 5); the mutant also showed defects in ethanol production on both glucose and xylose media—it produced 12.9 g/L of ethanol from glucose and 0.33 g/L of ethanol from xylose on 72 h of fermentation in comparison to respectively 23 g/L and 0.49 g/L by the WT strain (Fig. 6a, d, Table 1).

**Fig. 7:**
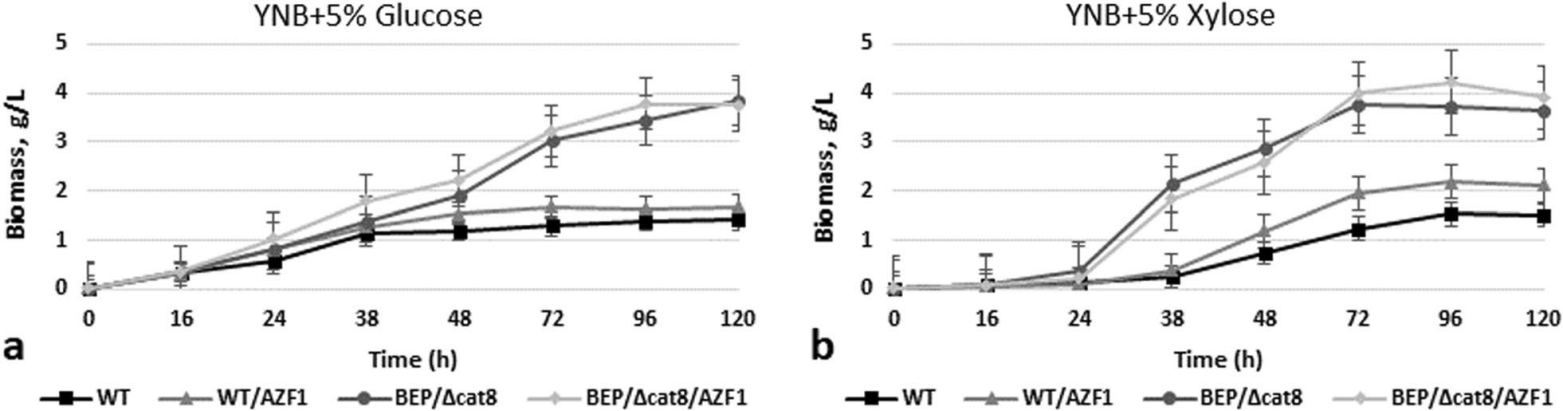
Growth test of the O. polymorpha wild type, BEP/∆cat8 and recombinant strains with *AZF1* gene overexpression on different media, cultivation at 37 °C

**Fig. 8:**
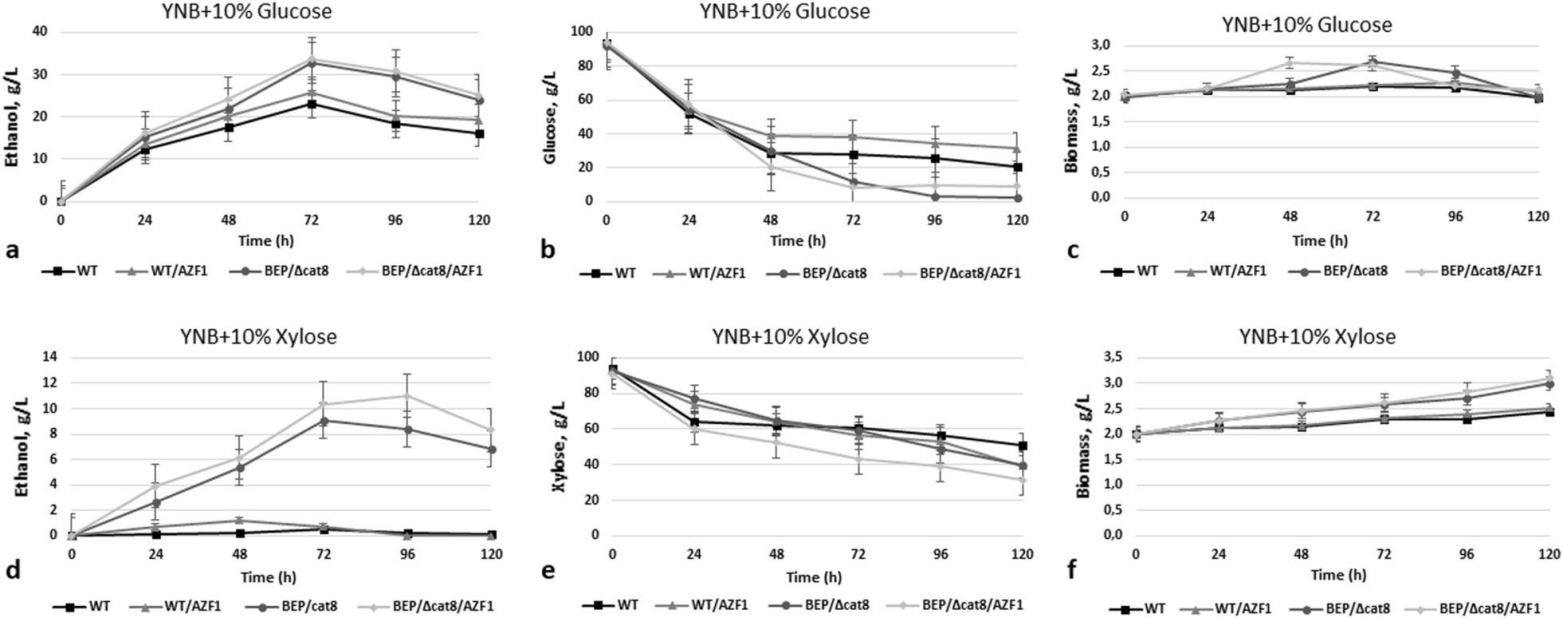
Ethanol production **a, d**, sugar consumption **b, e** and biomass accumulation **c, f** during 10% Glucose and 10% Xylose alcoholic fermentation of *O. polymorpha* wild type BEP/∆cat8 and recombinant strains with *AZF1* gene overexpression at 37 °C. Data are shown as mean of three independent experiments

Overexpression of the *AZF1* gene under the control of the *GAP1* gene promoter improved growth on both glucose and xylose relative to the parental strains (Fig. 7). *AZF1* overexpression activated alcoholic fermentation of xylose on background of the WT strain (approximately twice), as well as ethanol production from glucose. Its overexpression on background of the advanced ethanol producer from xylose (BEP/Δcat8) resulted in near 10% increase of ethanol accumulation on glucose and above 30% increase (up to 11 g/L on 72 h) in xylose medium (Fig. 8, Table 1), which are the promising results.

Ethanol yield, ethanol specific production rate and ethanol productivity during xylose alcoholic fermentation for all studied recombinant strains are also listed in Table 1. The highest ethanol yield of 354 mg per g of consumed xylose was observed in strain BEP/∆cat8/AZF1, whereas strain BEP/∆cat8/HXS1 has shown the highest ethanol specific production rate (63 mg/g biomass/h) and ethanol productivity (181 mg/L/h).

Constructed by us earlier recombinant mutant with activation of ethanol production from xylose BEP/∆cat8 [13] displayed, as expected, an increase in specific activities of xylitol dehydrogenase and pyruvate decarboxylase, relative the wild-type strain (Table 2). Overexpression of *HXS1* or *AZF1* on the background of such BEP/∆cat8 strain further elevated specific activities of xylitol dehydrogenase and pyruvate decarboxylase (Table 2). We suggest that an increase in ethanol production in the strains with overexpression of the *HXS1* and *AZF1* genes is a consequence of the observed elevation in activities of xylitol dehydrogenase and pyruvate decarboxylase. However, molecular mechanisms of the observed changes remain obscure and will be addressed in our future studies.

**Table 2:** Activities of the enzymes xylose reductase (**XR**), xylitol dehydrogenase (**XDH**), alcohol dehydrogenase (**ADH**) and pyruvate decarboxylase (**PDC**) in the tested *O.* *polymorpha* strains with the *HXS1* and *AZF1* genes deletion and overexpression. Activities were measured on 72 h of fermentation on xylose at 37 °C

Strain	Activity U/mg of protein			
	**XR**	**XDH**	**ADH**	**PDC**
NCYC 495 WT	0.99 ± 0.05	0.62 ± 0.03	0.97 ± 0.05	0.33 ± 0.02
WT/Δazf1	0.98 ± 0.04	0.64 ± 0.03	0.82 ± 0.03	0.43 ± 0.02
WT/AZF1	0.89 ± 0.04	0.50 ± 0.02	0.76 ± 0.03	0.53 ± 0.02
WT/Δhxs1	0.82 ± 0.04	0.56 ± 0.03	0.80 ± 0.04	0.52 ± 0.02
WT/HXS1	0.85 ± 0.04	0.67 ± 0.03	0.81 ± 0.03	0.37 ± 0.02
BEP/Δcat8	0.84 ± 0.04	1.05 ± 0.05	1.07 ± 0.05	0.69 ± 0.03
BEP/Δcat8/AZF1	0.87 ± 0.04	1.42 ± 0.06	0.70 ± 0.03	0.92 ± 0.05
BEP/Δcat8/HXS1	0.87 ± 0.04	1.47 ± 0.07	1.06 ± 0.05	0.83 ± 0.04

## Discussion

In this work we present data on the role of hexose sensor Hxs1 and transcription factor with sensing properties Azf1 on xylose and glucose fermentation in the native xylose-metabolizing yeast *O.* *polymorpha*. The role of native and heterologous hexose transporters in xylose metabolism and fermentation was recently studied [14]. The role of sugar sensors in xylose fermentation was also studied in xylose-metabolizing strains of *S. cerevisiae*. Positive effects of activation of the glucose sensing system in xylose-fermenting strains by upregulating the cAMP-PKA and Rgt2/Snf3-Rgt1 pathways were found [22]. In that work, it was shown that deleting cAMP phosphodiesterase genes *PDE1* and *PDE2* increased PKA activity in the strains, and consequently, increased xylose utilization. It was also hypothesized that *S. cerevisiae* mainly depends on Snf3 to sense high concentrations of xylose [22–24]. It is known that the intracellular glucose sensing in *S. cerevisiae* is mediated by hexokinase Hxk2. It was shown that expression of a nuclear-localized Hxk2 activated xylose fermentation [25]. Effects of engineering the Snf3/Rgt2, SNF1/Mig1 and cAMP/PKA signalling pathways on xylose metabolism and fermentation in *S. cerevisiae* have been recently reviewed [26]. The role of xylose sensing/signalling in xylose metabolism and fermentation in the natural xylose-utilizing yeasts remains poorly understood. Some studies in this direction have been done in *Scheffersomyces stipitis* and *Spathaspora passalidarum*, however, no specific sensing proteins involved in xylose alcoholic fermentation were identified [27, 28].

In the current work, the role of sugar sensors on xylose metabolism and fermentation was studied for the first time in the natural xylose-metabolizing yeast, *O. polymorpha*. Specifically, we evaluated the role of Hxs1 sugar sensor on glucose and xylose alcoholic fermentation in this species. It was found that this sensor is important not only for glucose utilization which was described before [15], but also for xylose metabolism and alcoholic fermentation of both sugars. Deletion of the *HXS1* gene strongly suppressed utilization and fermentation of both sugars whereas its overexpression activated these processes. It is important to note that overexpression of *HXS1* gene also enhanced ethanol production from xylose on background of the advanced ethanol producer from this pentose. The observed phenomenon, no doubt, is of great biotechnological significance.

The role of transcription factors in xylose (and glucose) alcoholic fermentation is relatively new field of research [29–31]. The important role of certain transcription factors in xylose and glucose alcoholic fermentation in *O.* *polymorpha* was found by us earlier. Thus, we have found significant and specific activation of xylose alcoholic fermentation due to knock out of the *CAT8* and *HAP4A* genes encoding for transcription activators whereas overexpression of the mentioned genes suppressed this process [10, 13]. At the same time, knock out of transcription activator genes *MIG1* and *MIG2* as well as knock out of transcription repressor gene *TUP1* strongly inhibited xylose alcoholic fermentation [10]. Herein we paid attention on the potential role of the homolog of transcription factor Azf1 which is known in *S. cerevisiae* to be involved in regulation of carbon source utilization and in glucose sensing [17, 32]. Besides, in xylose-utilizing strain of *S. cerevisiae*, Azf1 is required for efficient xylose fermentation [20]. In *O. polymorpha*, it was found that *AZF1* deletion retards both glucose and xylose fermentation, whereas overexpression of this gene, inversely, activates these processes. In the advanced ethanol producer from xylose [13], *AZF1* overexpression also elevates ethanol production from glucose and especially from xylose. These observations could be of biotechnological importance for construction of more efficient ethanol producers from lignocellulosic hydrolysates.

## Conclusions

The genes of the thermotolerant yeast *O. polymorpha* encoding for hexose sensor protein Hxs1 and for transcription factor involved in carbohydrate sensing Azf1 were found to be the promising tools for increasing ethanol production from both major lignocellulosic sugars, glucose and xylose.

## Methods

### Strains and growth conditions

*O. polymorpha* strains that were used in this study are listed in Table 3.

**Table 3:** Yeast strains that were used in this work

Strain name	Description	Source
WT	NCYC495 *leu1-1*	National Collection of Yeast Cultures (NCYC)
WT/LEU	NCYC495 *leu1-1*/*LEU2*	Obtained in this work on the background of WT strain
Ins	Insertional mutant with reduced ethanol production from xylose	Obtained in this work on the background of WT strain
WT/Δhxs1	NCYC495 *leu1-1 hxs1*Δ/*Zeo*^*R*^	Obtained in this work on the background of WT strain
WT/Δhxs1/LEU	NCYC495 *leu1-1 hxs1*Δ/*LEU2*	Obtained by our colleagues on the background of WT strain, in (15) named Hp015
WT/LEU/HXS1	NCYC495 *leu1-1*/*LEU2/HXS1/natNT*	Obtained in this work on the background of WT/LEU strain
WT/Δhxs1/LEU/HXS1	NCYC495 *leu1-1 hxs1*Δ/*LEU2/HXS1/natNT*	Obtained in this work on the background of strain WT/Δhxs1/LEU
WT/Δazf1	NCYC495 *leu1-1 azf1*Δ/*AUR1*	Obtained in this work on the background of WT strain
WT/AZF1	NCYC495 *leu1-1 AZF1/natNT*	Obtained in this work on the background of WT strain
BEP/Δcat8	NCYC495 *leu1-1*/2EtOH/XYL1m/XYL2/XYL3/BrPA/*cat8*Δ	Obtained in our previous works (11, 13)
BEP/Δcat8/HXS1	NCYC495 *leu1-1*/2EtOH/XYL1m/XYL2/XYL3/BrPA/*cat8*Δ*/HXS1/natNT*	Obtained in this work on the background of strain BEP/Δcat8
BEP/Δcat8/AZF1	NCYC495 *leu1-1*/2EtOH/XYL1m/XYL2/XYL3/BrPA/*cat8*Δ*/AZF1/natNT*	Obtained in this work on the background of strain BEP/Δcat8

Yeast strains were grown at 37 °C in a rich YPD medium (10 g/L yeast extract, 10 g/L peptone, 20 g/L or 40 g/L carbon source (glucose or xylose)) or mineral YNB medium (6.7 g/L YNB plus ammonium sulfate w/o amino acids, 20 g/L of carbon source). Leucine (40 mg/L) was added to the YNB medium for NCYC495 *leu1-1* strain and derivative recombinant strains.

The *E. coli* strain DH5a [*F80dlacZDM15, recA1, endA1, gyrA96, thi-1, hsdR17 (rK, mK 1), supE44, relA1, deoR, D (lacZYA-argF) U169*] was used in experiments that required a bacterial host. The bacterial strain was grown at 37 °C in a rich LB medium as was previously described [33]. Transformed *E. coli* cells were maintained on a LB medium containing 100 mg/L of ampicillin or 30 mg/L of zeocine.

### Molecular-biology procedures

Standard cloning techniques were carried out as described [33]. Plasmid and genomic DNA isolation, PCR-reactions were performed as was described in our previous works [11, 13, 14]. Transformation of the yeast *O.* *polymorpha* was carried out as described elsewhere [34].

### Vectors construction

For insertional mutagenesis, BamHI-linearized plasmid pPICZ-B (obtained from Nova lifetech Inc) was used. To obtain plasmid for sequencing of the regions flanking insertional cassette in the genome of Ins mutant, total genomic DNA was isolated from yeast cells, digested with the restriction endonuclease BglII, self-ligated and transformed into *E. coli* DH5a. Colonies were selected on a LB medium containing 30 mg/L of zeocine. From one of these colonies, 5062 bp-long plasmid was isolated containing insertion cassette together with flanking sequences derived from *O.* *polymorpha* genomic DNA. This plasmid, named pPICZ-B-Ins was used for sequencing of flanking sequences and for obtaining of the deletion mutant WT/Δhxs1.

Vector for deletion of the *AZF1* gene was constructed as following: genomic DNA of *O. polymorpha* NCYC495 strain was used as a template for PCR amplification of 5′ and 3′ non-coding regions of AZF1 using primers 5'AZF1F/5'AZF1R and 3'AZF1F/3'AZF1R (all used primers are listed in Table 4). Gene AUR1 conferring resistance to aureobasidin was amplified with primers plAUR1_F/plAUR1_R and united with 5' fragment of AZF1 by means of Gibson Assembly. As a result, plasmid plAUR1_5'AZF1 was obtained. At the next stage, plasmid plAUR1_5'AZF1 was amplified with primers plAUR1_5'AZF1_F/plAUR1_5'AZF1_R and combined with the 3' fragment of AZF1. The resulting vector was named pΔazf1.

**Table 4:** DNA oligonucleotides used in this study

Primer names	Primer sequence (5'–3')
5'AZF1F	GACGGCCAGTGCTACCATATCTCAATCAAC
5'AZF1R	CGCGAATTCCATTTCCAGGGTCTGGGTG
3'AZF1F	GGCACGAATTCCAGAGGAGTGAAAAACGCAG
3'AZF1R	GGTACCGAGCTCCAAGTTGGAAAGTCTCAATTC
plAUR1_F	CTGGAAATGGAATTCGCGCAATAGGGTCTC
plAUR1_R	GGTAGCACTGGCCGTCGTTTTACAAC
plAUR1_5’AZF1_F	CAACTTGGAGCTCGGTACCTCGCGAATG
plAUR1_5’AZF1_R	CTCCTCTGGAATTCGTGCCTAATCAAATGCATTC
SM166	TGCTCTAGAATGTCGACAGAAGCTCGAGATCAc
SM167	TTTGCGGCCGCTTATTGTCCATGGCTATGTACAGACTCTG
AZF1_F	AAAGCGGCCGCATGCCGATGATATACTC
AZF1_R	AAAGCGGCCGCTCAAACTCCATGACCG
SM7	CCCTCACATGCAGAACTAACCAATAAGG
RV130	TCATTATCATATACCGCAGCGC
RV140	CAAGCAGAGTGCAGAGATGG
Ko1036	AGTCATACGTGTAGGTTTTTGGCG
RV131	GCGCAATAGGGTCTCTTTC
RV132	GCATTAATTGATCCACCTTTG

Vectors for overexpression of the genes *HXS1* and *AZF1* were constructed as described below. *O.* *polymorpha* genome database (https://mycocosm.jgi.doe.gov/Hanpo2/Hanpo2.home.html) was used for retrieval of the *HXS1* and *AZF1* genes sequences*.* Vector pUC19-GAPpr-GAPt-natNT2 was obtained as described in [35]. 1917 bps long DNA fragment corresponding to the ORF of the gene *HXS1* was amplified from the genomic DNA of NCYC495 strain using primers SM166 and SM167, digested with restriction endonucleases XbaI and NotI, and cloned into the corresponding sites of the plasmid pUC19-GAPpr-GAPt-natNT2. The resulting vector was named pHXS1. 1911 bp DNA fragment corresponding to the ORF of the gene *AZF1* was amplified from the genomic DNA of NCYC495 strain using primers AZF1_F/AZF1_R, digested with restriction endonuclease NotI and cloned into the corresponding site of the pre-dephosphorylated pUC19-GAPpr-GAPt-natNT2 vector. The resulting vector was named pAZF1.

### Selection of *O. polymorpha* transformants

To obtain the deletion mutant WT/Δhxs1, vector pPICZ-B-Ins was digested with the restriction endonuclease BglII and used for transformation of *O. polymorpha* strain NCYC495. Transformants were selected on a solid YPD medium supplemented with 150 mg/L of zeocine. Deletion of the *HXS1* gene was verified using primers SM7/SM167.

To obtain the deletion mutants WT/Δazf1, linearized vector pΔazf1 was transformed into *O.* *polymorpha* NCYC495 as a recipient strain. Transformants were selected on the solid YPD medium supplemented with 0.3 g/L of aureobasidin after 4 days of incubation. Deletion of the *AZF1* gene was verified using primers RV130/RV140.

To obtain recombinant strains with *HXS1* or *AZF1* overexpression, vectors pHXS1 and pAZF1 were digested with the restriction endonuclease BamHI and used for transformation of *O. polymorpha* strains NCYC495 and BEP/Δcat8. Transformants were selected on a solid YPD medium supplemented with 150 mg/L of nourseothricin. The selected transformants were stabilized by alternating cultivation in non-selective and selective media and examined by diagnostic PCR using primers Ko1036/SM167 for pHXS1 vector and RV131/RV132 for pAZF1 vector.

### Ethanol production and sugars consumption assays

Alcoholic fermentation of yeast strains was performed by cultivation in liquid mineral medium at oxygen-limited conditions at 37 °C. The cells were pre-grown in 100 mL of liquid YPD or YPX medium (10 g/L yeast extract, 20 g/L peptone and 40 g/L glucose or xylose, respectively) in 300 mL Erlenmeyer flasks at 220 rpm till the mid-exponential growth phase. Then the cells were harvested by centrifugation, washed by water and inoculated into 40 mL of the fermentation medium (containing 6.7 g/L YNB plus ammonium sulfate w/o amino acids and leucine, if necessary) to the final cell density of 2 mg of dry weight/mL. 100 g/L glucose or xylose was added into the mineral medium used for fermentation. The oxygen-limited conditions were provided by agitation at 140 rpm.

Samples of medium for ethanol production assay were taken each day. The biomass was determined turbidimetrically with a Helios Gamma spectrophotometer (OD, 590 nm; cuvette, 10 mm) with gravimetric calibration. Concentrations of glucose, xylose and ethanol in medium broth were analyzed by HPLC (Perkin Elmer, Series 2000, USA) with an Aminex HPX-87H ion-exchange column (Bio-Rad, Hercules, USA). A mobile phase of 4 mM H_2_SO_4_ was used at a flow rate 0.6 mL/min and the column temperature was 35 °C. Alternatively, the concentration of ethanol in the medium was determined using the “Alcotest” kit [36]. Fermentation experiments were performed at least in duplicate.

### Biochemical methods

Samples for enzyme activity assays were taken from the cultures on the third day of xylose fermentation at 37 °C. The enzyme activity was measured directly after the preparation of cell-free extracts. Protein concentration was determined with Folin reagent [37]. The specific activities of XR and XDH were determined spectrophotometrically as described before [8]. The PDC activity in cell extracts was determined spectrophotometrically according to the method described earlier [38]. The ADH activity was measured by following the reduction of NAD at 340 nm using 96% ethanol as a substrate as described previously [13].

### Statistical analysis

All the experimental data shown in this manuscript were collected from three independent samples to ensure reproducibility of the trends and relationships observed in the cultures. Each error bar indicates the standard deviation (SD) from the mean obtained from triplicate samples. The 5% significance level was used in the statistical analyses.

## Data Availability

All data generated or analyzed during this study are included in this published article. Generated strains and vectors are available from the corresponding author on reasonable request.
